# Redescription of the Far Eastern brook lamprey *Lethenteron reissneri* (Dybowski, 1869) (Petromyzontidae)

**DOI:** 10.3897/zookeys.506.9817

**Published:** 2015-05-28

**Authors:** Claude B. Renaud, Alexander M. Naseka

**Affiliations:** 1 Research & Collections Division, Canadian Museum of Nature, P.O. Box 3443, Station D, Ottawa, ON K1P 6P4 Canada; 2 Faculty for Biology and Soil, St. Petersburg State University, Universitetskaya Emb. 7, St. Petersburg, 199034 Russia

**Keywords:** Amur River basin, morphology, nonparasitic, taxonomy

## Abstract

Nonparasitic *Lethenteron reissneri* (Dybowski) is redescribed based on four syntypes (two adults and two ammocoetes) from the Onon and Ingoda rivers, Russia, and 15 topotypic specimens (seven metamorphosing ammocoetes and eight ammocoetes) from the Onon River system, Russia and Mongolia. Posterial teeth were not mentioned in the original description, but Berg (1931) stated that they were sometimes absent, which he later (Berg 1948) changed to usually absent, based on material (some of which we have re-identified as parasitic *Lethenteron camtschaticum*) from far outside of the type locality. The latter view has been widely accepted by subsequent authors. Unfortunately, the poor condition of the two adult syntypes did not permit verification of this character. However, a row of posterials was clearly visible in six of the seven topotypic metamorphosing ammocoetes and indicates their usual presence in the species. The first full description of the ammocoetes, including pigmentation, is provided. The present study restricts the distribution of *Lethenteron reissneri* to the Shilka and Songhua river systems within the Amur River basin, until a more geographically comprehensive study is undertaken. Additionally, in this study, feeding versus non-feeding at the adult stage, are considered to be valid taxonomic characters at the species level.

## Introduction

The Northern Hemisphere lamprey genus *Lethenteron* was originally erected as a subgenus of *Entosphenus* by Creaser and Hubbs (1922) and elevated to generic rank by Jordan et al. (1930), without justification. Vladykov and Follett (1967) accepted this action and defined members of *Lethenteron* as possessing posterial teeth in a single curved row and with the outer laterals (exolaterals) absent. Renaud (2011) recognized one parasitic species, *Lethenteron camtschaticum* (Tilesius, 1811) (i.e., the stem species) and six nonparasitic species, *Lethenteron alaskense* Vladykov & Kott, 1978, *Lethenteron appendix* (DeKay, 1842), *Lethenteron kessleri* (Anikin, 1905), *Lethenteron ninae* Naseka, Tuniyev & Renaud, 2009, *Lethenteron reissneri* (Dybowski, 1869), and *Lethenteron zanandreai* (Vladykov, 1955) (i.e., the satellite species) in the genus, along with two additional undescribed nonparasitic species from Japan, and noted that the taxonomic limits of the nonparasitic *Lethenteron reissneri* were unclear. Authors have either treated these satellites of *Lethenteron camtschaticum* as distinct species (e.g., Potter et al. 2015) or as synonyms of this species. For example, Artamonova et al. (2011a, 2011b) and Makhrov et al. (2013) have, respectively, suggested that *Lethenteron ninae*, *Lethenteron reissneri*, and *Lethenteron kessleri* are synonyms of *Lethenteron camtschaticum* and that mode of life (i.e., parasitism versus nonparasitism) is not a valid criterion for specific distinctiveness. This is not a new concept. McPhail and Lindsey (1970), in discussing *Lethenteron camtschaticum* (reported as *Lampetra japonica* (von Martens, 1868)) from the Northwest Territories, Yukon and Alaska, suggested that it may not be specifically distinct from *Lethenteron appendix* (reported as *Lethenteron lamottenii* (Lesueur, 1827)) from eastern North America, but preferred to retain them as distinct pending a review of all relevant species from both continents (i.e., North America and Eurasia). Renaud et al. (2009) proposed that the key to resolving the issue of whether parasitism or nonparasitism constitute valid taxonomic characters at the species level may be to conduct common garden experiments (i.e., rearing two putative species under the same conditions from the zygote to the adult stage) that seek to elucidate the triggering mechanism for trophic interactions, or the lack thereof, in the adult stage. In the absence of such experiments, however, we follow here conventional taxonomy and continue to recognize the above nonparasitic species as distinct. Additionally, prior to synonymizing species, it is critical to compare any extant type material as well as the original descriptions, and, unfortunately, this has not been done on a consistent basis by authors. The original description by Dybowski (1869) of *Petromyzon reissneri* was very short, incomplete and based on material from the upper Amur River basin, Russia (type locality: Onon and Ingoda rivers). A number of authors conducted morphological studies on what they referred to as *Lampetra reissneri* and anadromous *Lethenteron japonica* (= *Lethenteron camtschaticum*) from the lower Amur River basin (Berg 1931, 1948, Nikol’sky 1956, Abakumov 1960, Hensel 1963). However, we are aware of only two studies since the original description, to have examined lamprey material, which they treated as a single species, from the upper Amur (Karasev 1987, Yamazaki et al. 2006). The Amur River basin is divided into five or six well-defined zoogeographic districts (Bogutskaya et al. 2008) or ecoregions (Abell et al. 2008), respectively, based on the distributional disparities of freshwater fish species along its 4,370–4,510 km length. Thus, it is critical to examine lamprey material from the upper rather than lower Amur, to objectively evaluate the characteristics of *Lethenteron reissneri* and prevent the inclusion of another species in the redescription. Karasev (1987) studied 52 adult lamprey from the Ingoda and the Shilka rivers, but only reported a few morphometric characters and the tooth formula of the infraoral lamina. Yamazaki et al. (2006) studied 26 ammocoetes from the Onon and 29 from the Ingoda rivers and determined that they possessed 65–73 trunk myomeres, but made no mention of their body pigmentation. Yamazaki et al. (2006) examined an adult syntype of *Lethenteron reissneri* (Museum für Naturkunde, Berlin; ZMB 7118) and determined it had 69 trunk myomeres, but made no mention of its dentition. Although Berg (1931) reported that *Lethenteron reissneri* possessed 56–67 trunk myomeres, that range was taken from Jordan and Hubbs (1925) based on Japanese material (Himeji and Lake Biwa, Honshu Island and Sapporo, Hokkaido Island), which they identified as *Entosphenus mitsukurii* (Hatta 1901). Berg (1931) further stated that the lower labial teeth (= posterial teeth) were sometimes absent in *Lethenteron reissneri*. He subsequently (Berg 1948) changed this to usually absent based on material from the Asian Pacific coast between the Anadyr estuary and the Shangshi River, distant from the type locality. Hubbs and Potter (1971) highlighted the inconsistency between Berg’s (1948) statement of usually no lower labial teeth and their observations. Nevertheless, Reshetnikov (2002) repeated Berg’s (1948) statement in the widely cited Atlas of freshwater fishes of Russia. The purpose of this study is to provide a full description of the species based on the examination of type and topotypic material of ammocoetes, metamorphosing ammocoetes and adults, and in view of the inconsistency between the observations of Berg (1931, 1948) and Hubbs and Potter (1971), re-examine material that Berg studied to determine how often posterials are absent.

## Materials and methods

Material was examined from the Academy of Natural Sciences, Philadelphia (ANSP), Natural History Museum, London (BMNH), Canadian Museum of Nature Fish Collection, Ottawa (CMNFI), Naturhistorisches Museum Wien, Vienna (NMW), Zoological Institute, Russian Academy of Sciences, St. Petersburg (ZIN), and Museum für Naturkunde, Berlin (ZMB). Note that collection dates for ZIN collections use the Gregorian rather than the Julian calendar. The characters examined in the ammocoetes and adults follow the method of Renaud (2011). Evaluation of the lateral line neuromast pigmentation in both ammocoetes and adults was made on the dorsal aspect of the branchial region. We additionally examined in ammocoetes the pigment pattern of the posterior fleshy part of the tail as in Richards et al. (1982); their caudal ridge, and the pigmentation in adults of the gular region and apex of the second dorsal fin following Vladykov and Kott (1978). TL, total length.

**Figure 1.**
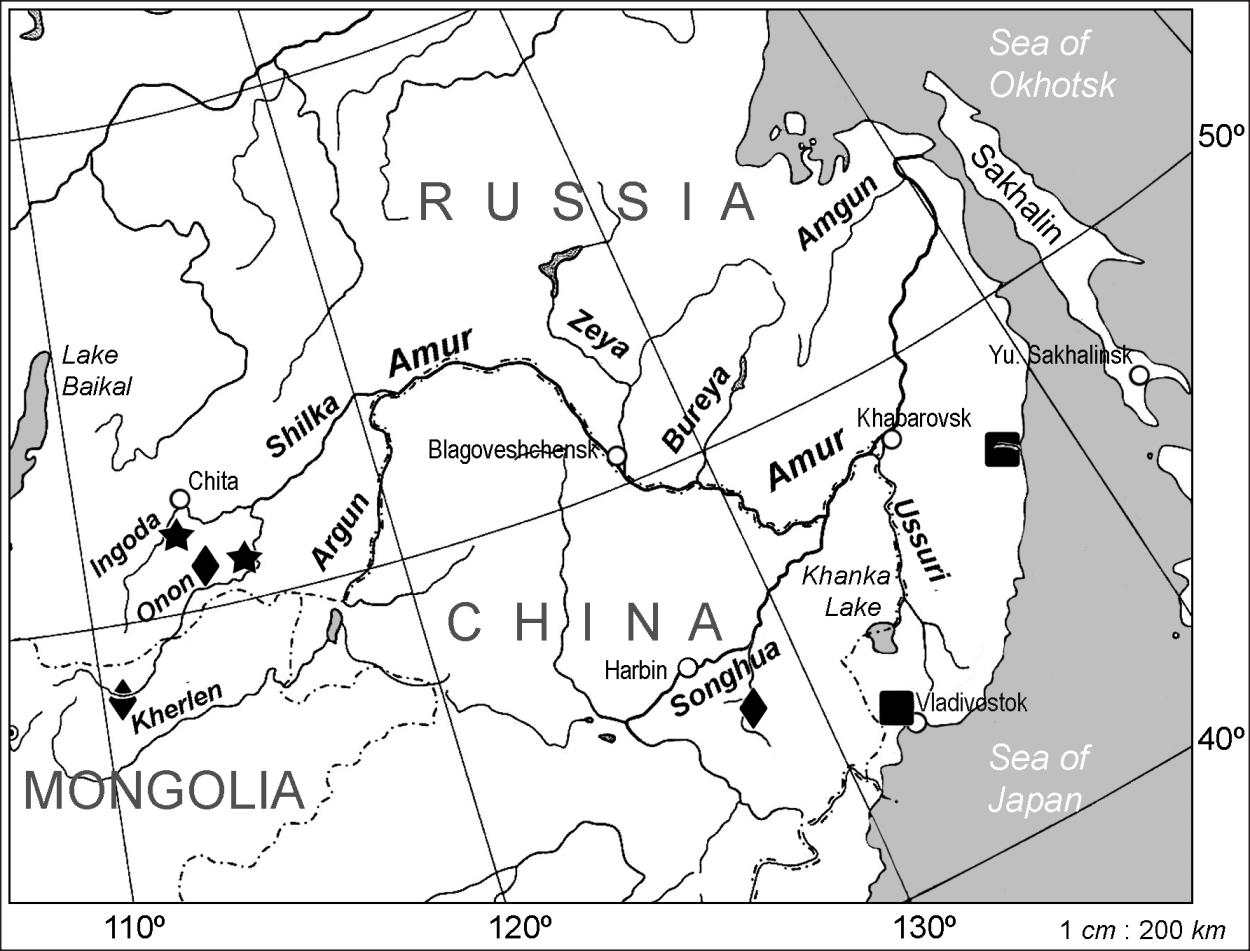
Geographic distribution of *Lethenteron reissneri* and material identified by Berg (1931) as *Lethenteron reissneri* without posterials. Approximate location of the type locality of *Lethenteron reissneri*, Amur River basin, Russia (solid star), topotypic localities in Russia and Mongolia (solid diamonds) and localities of material identified by Berg (1931) as *Lethenteron reissneri*: Shangshi River, Amur River basin, People’s Republic of China (solid diamond), Sedanka River, Sea of Japan basin, Russia (solid square), and Samarga River, Sea of Japan basin, Russia (solid square). The Sedanka and Samarga River specimens were re-identified as *Lethenteron camtschaticum*.

**Syntypes** (for locality see also map in Fig. 1)

BMNH 1871.7.19.37, 1 adult, reported as 120 mm TL in Regan (1911), but only 90+ mm when measured by CBR in 2010, Russia: Onon River, Transbaikalia (also known as Dauria), purchased from Museum Godeffroy (Museum Godeffroy, Hamburg, Germany, existed 1861 to 1885); ZMB 7118, 1 adult, 117.1 mm TL, Russia: Onon River, B.N. Dybowski; NMW 78112, 2 ammocoetes, 106.5–122.2 mm TL, Russia: Onon and Ingoda rivers, upper Amur River basin, received from B.N. Dybowski on 1 Feb. 1870.

**Topotypic non-type material** (for localities see also map in Fig. 1)

ANSP 185410, 2 ammocoetes, 83–151 mm TL, 6 metamorphosing ammocoetes, 139.5–151.5 mm TL, Mongolia: Barh River, tributary to Onon River, upper Amur River basin, about 5 km E of town of Barh at crossing of road N to Bathshireet, Khentii Province, altitude 1,111 m, 48°36.20'N; 110°12.00'E, 21 Aug. 2006; CMNFI 2008–58, 1 metamorphosing ammocoete, 164 mm TL, Russia: Ilya River, tributary to Onon River, upper Amur River basin, upstream from Dul’durga, 50°43.18'N; 113°35.42'E, 24–26 Aug. 2004; ZIN uncatalogued, 6 ammocoetes, 129.5–171 mm TL, Russia: Ilya River, tributary to Onon River, upper Amur River basin, upstream from Dul’durga, 50°43.18'N; 113°35.42'E, 24–26 Aug. 2004.

**Specimens identified by Berg (1931) as *Lethenteron reissneri*** (for localities see also map in Fig. 1)

ZIN 14457, 1 adult female, 146 mm TL, 1 ammocoete, 221.5 mm TL, People’s Republic of China: Shanshi (= Shangshi) River, Sungari (= Songhua) River system, at Khandaokhetszy railway station, near Shangzhi, Manchuria, April 1908; ZIN 15078, 1 adult re-identified as *Lethenteron camtschaticum*, 178+ mm TL (very dessicated), Russia: Samarga River, near Sufren Cape, near coast, in brackish water, 28 Sept. 1910; ZIN 15747, 1 adult female re-identified as *Lethenteron camtschaticum*, 161.5 mm TL, Russia: Sedanka River, near Vladivostok, 6 March 1912.

## Results

**Adults** (Tables 1 and 2)

**Table 1.** Morphometrics re-examined in adult *Lethenteron reissneri* and *Lethenteron camtschaticum* (identified by Berg (1931) as *Lethenteron reissneri*). The syntypes and non types of *Lethenteron reissneri* are from upper Amur River basin.

	*Lethenteron reissneri* syntype† BMNH 1871.7.19.37 Onon River	*Lethenteron reissneri* syntype ZMB 7118 Onon River	*Lethenteron reissneri* non types (from Karasev 1987) Ingoda and Shilka rivers n = 52	*Lethenteron reissneri*	*Lethenteron camtschaticum* specimens identified by Berg (1931) as *Lethenteron reissneri*	
				ZIN 14457 Shangshi River	ZIN 15747 Sedanka River	ZIN 15078 Samarga River
Total length (TL, mm)	90.0+	117.1	137–182	146.0	161.5	178.0+
%TL Prebranchial length	undetermined	11.1	7.9–15.0	11.0	9.9	10.4
Branchial length	undetermined	10.9	8.9–11.2	10.3	9.3	9.6
Trunk length	undetermined	50.5	undetermined	47.9	52.0	49.4
Cloacal slit length	undetermined	1.2	undetermined	0.7	0.9	1.1
Tail length	undetermined	26.9	undetermined	28.8	28.2	27.8
Disc length	3.9	5.1	undetermined	4.8	4.3	4.8
Snout length	undetermined	6.1	4.0–9.3	6.8	5.0	5.9
Eye length	2.8	1.5	1.2–2.2	2.1	2.5	1.7
Postocular length	undetermined	3.5	2.2–3.9	2.7	2.5	2.2
Interbranchial opening length	undetermined	1.5	undetermined	1.7	1.2	1.1
Prenostril length	undetermined	4.9	undetermined	5.1	4.6	5.1
Interocular width	undetermined	3.0	2.2–3.9	3.8	3.1	2.2
Urogenital papilla length	undetermined	0.9	undetermined	0.7	0.6	undetermined

† Extremely dessicated and contorted specimen; total length cannot be determined accurately.

**Table 2.** Characteristics re-examined in adults of *Lethenteron reissneri* (syntypes) and those identified by Berg (1931) as that species. Pigmentation coverage as follows: - < 1%; +++ ≥ 75%. b, bicuspid; u, unicuspid.

	*Lethenteron reissneri* syntype BMNH 1871.7.19.37 Onon River	*Lethenteron reissneri* syntype ZMB 7118 Onon River	*Lethenteron reissneri*	*Lethenteron camtschaticum* specimens identified by Berg (1931) as *Lethenteron reissneri*	
			ZIN 14457 Shangshi River	ZIN 15747 Sedanka River	ZIN 15078 Samarga River
Trunk myomeres	undetermined	70	72	72	77
Supraoral lamina	1u–1u	1u–1u	1u–1u	1u–1u	1u–1u
Endolaterals	undetermined	2–2–2	2–2–2	2–2–2	2–2–2
Infraoral lamina	undetermined	1b5u	1b4u1b	1b4u1b	6u1b
Rows of anterials	undetermined	undetermined	3	2	2
Rows of exolaterals	undetermined	undetermined	0†	0	0§
Rows of posterials	undetermined	undetermined	2	1	1
First anterial row	undetermined	undetermined	5u	5u	4u
First posterial row	undetermined	undetermined	24u	18u	18u1b3u
Transverse lingual lamina	undetermined	undetermined	2u–I–2u	4u–I–4u	4u–I–4u
Longitudinal lingual laminae	undetermined	undetermined	undetermined	undetermined	undetermined
Oral papillae	undetermined	undetermined	undetermined	21	≈18
Oral fimbriae	undetermined	undetermined	≈104	92	91
Caudal fin shape	undetermined	undetermined	spade-like	spade-like	undetermined
Pigmentation - Caudal fin	undetermined	undetermined	+++	+++	+++
- Second dorsal fin	undetermined	undetermined	no blotch	with blotch	undetermined
- Lateral line neuromasts	undetermined	undetermined	unpigmented	unpigmented	undetermined
- Gular	undetermined	undetermined	-	-	-

† Left side has two exolateral teeth; one between first and second and other between second and third endolaterals.

§ Right side with exolateral tooth between first and second endolaterals.

**Figure 2.**
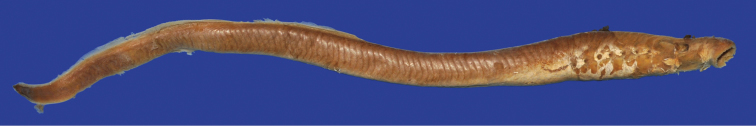
Syntype (adult) of *Petromyzon reissneri* Dybowski, 1869, ZMB 7118, 117.1 mm TL, Onon River.

Adult syntype (BMNH 1871.7.19.37) extremely dessicated and TL could not be accurately determined. Approximate TL 90+ mm. Eye length 2.5 mm. Confirmed as adult because of oral disc about 3.5 mm in length. Supraoral lamina with two cusps separated by broad toothless bridge. Two dorsal fins. No other counts or measurements feasible. Second adult syntype (ZMB 7118, Fig. 2) in much better condition and all morphometrics determined (Table 1) plus additional characteristics of dentition and trunk myomeres (Table 2). 70 trunk myomeres in syntype (69 reported by Yamazaki et al. (2006) for same specimen).

**Metamorphosing ammocoetes**

Seven topotypic metamorphosing ammocoetes from two tributaries to the Onon River examined: six from Barh River, Mongolia (ANSP 185410) and one from Ilya River, Russia (CMNFI 2008–58). Five of Mongolian specimens (139.5–151.5 mm TL) had remnants of oral cirrhi, whereas none present in the other Mongolian (147 mm) or the Russian (164 mm) specimens. Furrow linking all branchial openings present in all specimens except only partially present in one Mongolian specimen (146.5 mm). All specimens with both well-developed supraoral lamina bearing two cusps separated by wide bridge and faint dark blotch near apex of their second dorsal fin. Row of posterial teeth discernible in six specimens; although exact numbers could not be determined. This character not assessed in 139.5 mm Mongolian specimen because of presence of remnants of oral cirrhi on posterior field. Enlarged median cusp present on transverse lingual lamina in all specimens.

**Ammocoetes** (Table 3)

**Table 3.** Characteristics in ammocoetes of *Lethenteron reissneri* from upper Amur River basin and Shangshi River. Pigmentation coverage is as follows: -, absent to trace; +, 1% to < 25%; ++, 25% to < 75%; +++, ≥ 75%. The number in parentheses indicates the number of specimens with this condition.

	syntypes NMW 78112 n = 2	topotypes ANSP 185410 n = 2	topotypes ZIN uncatalogued n = 6	Shangshi River ZIN 14457 n = 1
Total length (TL, mm)	106.5–122.2	83.0–151.0	129.5–171.0	221.5
Prebranchial length (% TL)	6.3–6.9	7.0–8.4	5.8–7.3	5.0
Branchial length (% TL)	10.5–11.1	11.9–12.7	11.1–13.1	10.8
Trunk length (% TL)	55.9–57.7	50.6–53.6	49.8–53.3	53.7
Cloacal slit length (% TL)	0.5–0.7	0.6–1.0	0.6–1.9	1.4
Tail length (% TL)	24.0–24.5	26.5–29.1	27.5–28.8	29.1
Prenostril length (% TL)	1.6–2.1	2.0–2.4	1.7–2.4	1.8
Interbranchial opening length (% TL)	1.3–1.6	2.0–2.4	1.7–2.3	1.4
Trunk myomeres	67–69	67–69	66–70	≈70
Caudal fin shape	spade-like(2)	spade-like(2)	spade-like(6)	spade-like
Pigmentation - Upper lip	undetermined	+(2)	+(6)	-
- Lower lip	undetermined	++(2)	+(4), ++(2)	-
- Between upper lip and cheek	undetermined	+++(2)	+++(6)	+++
- Cheek	undetermined	+++(2)	+++(6)	++
- Subocular	undetermined	-, +	-, +(4), ++	-
- Upper prebranchial	undetermined	++(2)	+, ++, +++(4)	-
- Lower prebranchial	undetermined	-, +	-(3), +(3)	-
- Upper branchial	undetermined	++(2)	++(5), +++	++
- Lower branchial	undetermined	-(2)	-(6)	-
- Ventral branchial	undetermined	-(2)	-(6)	-
- Caudal fin	undetermined	++(2)	+(2), ++(4)	++
- Posterior fleshy part of tail	dark midline streak(2)	dark midline streak(2)	undetermined	dark midline streak
- Predorsal	undetermined	+, ++	+++(6)	++
- Lateral line neuromasts	undetermined	unpigmented(1), undetermined(1)	unpigmented(6)	unpigmented
Middle prong of tongue precursor - shape	undetermined	undetermined	undetermined	bulbous
- pigmentation	undetermined	undetermined	undetermined	++
Areas lateral to elastic ridge pigmentation	undetermined	undetermined	undetermined	-

**Figure 3.**
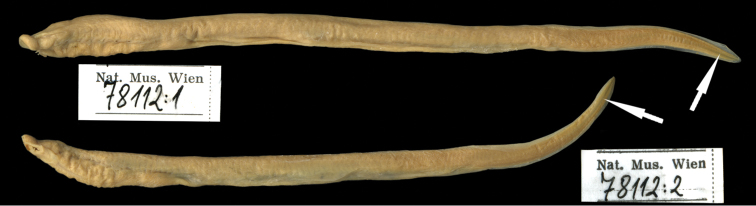
Syntypes (ammocoetes) of *Petromyzon reissneri* Dybowski, NMW 78112, 106.5 mm (below); 122.2 mm TL (above). Arrows point to dark midline streak on tail.

Morphometrics and number of trunk myomeres were determined in two syntypic ammocoetes (NMW 78112, Fig. 3), but specimens extremely faded with virtually no pigmentation. Both possessed prominent streak of dark pigmentation along midline of tail region (Fig. 3). This limited information was augmented by the study of eight topotypic ammocoetes (ANSP 185410 and ZIN uncatalogued) in which virtually all characters studied were recorded.

## Discussion

In the original description of *Petromyzon reissneri* written in German, Dybowski (1869) did not give the number of adults examined and the diagnosis was: 120–140 mm in TL; supraoral lamina with one blunt tooth at each end; infraoral lamina with six blunt teeth, the lateralmost being bicuspid; second dorsal fin almost three times higher than the first dorsal fin and higher than the body depth; dorsal body surface dark and lower surface whitish. Additional information was that spawning occurs in June and that ammocoetes are widespread and abundant, while adults are extremely rare. The small size of adults, the very high second versus first dorsal fins, and the strong bicoloration of the body points to the diagnosis being based on spawning individuals.

### Absence vs. presence of posterials in *Lethenteron reissneri*

Dybowski (1869) did not mention posterials in his brief original description of *Lethenteron reissneri*. Regan (1911) examined a 120 mm adult syntype from the Onon River (BMNH 1871.7.19.37) and identified it as *Lampetra planeri* (Bloch, 1784), a species that does not possess posterial teeth according to the key he provided in the same publication. Unfortunately, the condition of this syntype, as well as the one in Berlin (ZMB 7118), was such that the presence or absence of posterials could not be ascertained by us. However, the presence of a row of posterials in all topotypic metamorphosing ammocoetes in which this character was discernible, albeit of uncertain numbers, showed that posterials are the predominant if not constant characteristic of this species. This contrasts with the assumption in the recent literature (e.g., Reshetnikov 2002) that posterials in *Lethenteron reissneri* are sometimes (Berg 1931) or usually (Berg 1948) absent.

**Figure 4.**
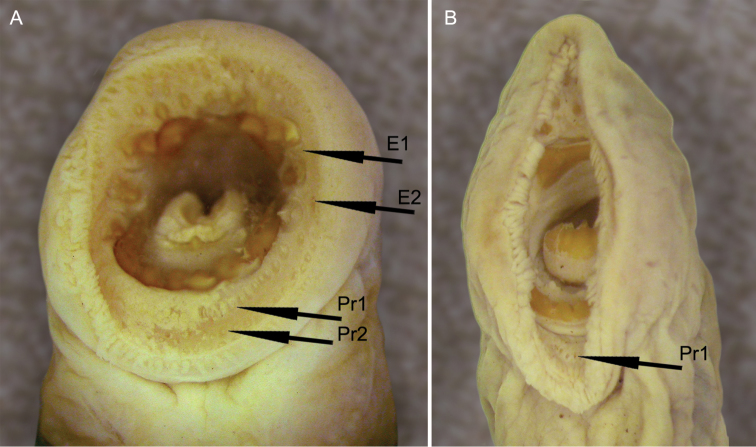
Oral disc of lampreys identified by Berg (1931) as *Lethenteron reissneri* without row of posterials. **A** ZIN 14457, Shangshi River, Songhua (Sungari) River system, People’s Republic of China, 146 mm TL with complete posterial row comprising 24 unicuspid teeth (Pr1; only few of these teeth visible in photo). Note second row of posterials (Pr2). Two exolateral teeth additionally present on left side, one between first and second (E1) and other between second and third endolaterals (E2) **B** ZIN 15078 (re-identified as *Lethenteron camtschaticum*), Samarga River, near Sufren Cape, Russia, 178.0+ mm TL with complete posterial row comprising 18 unicuspid, 3 bicuspid, 1 unicuspid teeth (only few of these teeth visible in photo). Exolateral tooth present on right side between first and second endolaterals not visible because oral disc not fully open.

Berg (1931) did not examine any material from the type locality, but recognized *Lethenteron reissneri* as a distinct species. He stated that in this species, the posterial teeth (he called them lower labial or infralabial teeth) were either entirely absent or present as a complete row and he provided drawings of the oral disc, showing these respective conditions [Berg (1931): Pl. VI, Fig. 2, ZIN 15747 and Pl. VI, Fig. 3, ZIN 15734] in two specimens from the Sedanka River, Russia. He documented five cases of *Lethenteron reissneri* without posterials (ZIN 14457, 15078, 15547, 15747, and an uncatalogued male from the Anadyr River). We were able to locate three of these specimens (ZIN 14457, 15078, 15747). Our re-examination revealed the following counts of posterials in a complete row: 24 unicuspid teeth (Fig. 4a); 18 unicuspid, 3 bicuspid, 1 unicuspid teeth (Fig. 4b); and 18 unicuspid teeth (Table 2). In fact, ZIN 14457 (Shangshi River, People’s Republic of China) possessed two rows of posterials (Fig. 4a). It is unknown why these numerous posterial teeth were overlooked by Berg; however, it is clear that an absence of posterials in the drawings of the oral disc [Berg (1931): Pl. VII, Fig. 1, ZIN 14457 and Pl. VI, Fig. 2, ZIN 15747] is incorrect. Berg (1948) partially corrected his 1931 comment by stating that non-keratinized posterials could be seen on the left side of the oral disc of specimen ZIN 14457 at 20× magnification. Based on its September collection date, strong dentition with nine teeth on the transverse lingual lamina and the fact that it was collected from brackish waters, ZIN 15078 (Fig. 4b) from the Samarga River, Russia was re-identified as a young adult *Lethenteron camtschaticum* on its feeding migration to the sea. The specimen from the Sedanka River (ZIN 15747) was likewise re-identified as *Lethenteron camtschaticum* based on the presence of nine strong teeth on its transverse lingual lamina. However, we agree with Berg (1931) that the adult from the Shangshi River, Songhua River system (ZIN 14457) is *Lethenteron reissneri* on the basis of its weaker dentition and lower number of teeth (i.e., five) on its transverse lingual lamina. Additionally, the *Lethenteron reissneri* specimen from the Shangshi River has an unpigmented second dorsal fin whereas the *Lethenteron camtschaticum* from the Sedanka River has a blotch on this fin (Table 2).

Karasev (1987) reported on seven morphometric characters comparable to those we examined (Table 1) along with the tooth formula of the infraoral lamina in 52 adults from the Ingoda and Shilka rivers in the upper Amur River basin. His morphometric data are informative in encompassing a range of adult total lengths (137–182 mm). The values recorded in the six morphometric characters that were expressed as percentages of TL for the best preserved syntype (ZMN 7118, 117.1 mm, Table 1) fall within the ranges reported by Karasev (1987). Unfortunately, it is unclear from the reported infraoral lamina counts (i.e., 1–4–1 and 1–5–1) whether the lateralmost teeth were enlarged relative to the internal ones. Regardless, the total number of teeth (6–7) is very similar to the only infraoral lamina count (6) in a syntype (ZMB 7118, Table 2). Karasev (1987) observed two lampreys attached to specimens of *Leuciscus waleckii* (Dybowski, 1869) in the Olengui River (tributary to the Ingoda River) in June 1960 and noted these were the only cases of lamprey attacking fishes in rivers of Transbaikalia observed over a period of years and that an unspecified number of examined adults had empty intestines. We do not consider these observations evidence of lamprey parasitism because attachment does not necessarily lead to feeding and especially since June, as noted above, is the spawning period in the upper Amur River basin and lampreys do not feed during this period (Potter et al. 2014). We believe additionally, that the small size difference between the largest ammocoetes (ZIN uncatalogued, 171 mm TL, Table 3) and adults [182 mm TL, Karasev (1987)] from the upper Amur River system is not conclusive evidence that *Lethenteron reissneri* feeds post metamorphosis and we presume that ammocoetes exceeding 182 mm TL exist. In fact, the 221.5 mm TL ammocoete and 146 mm TL spawning female from the Shangshi River, Songhua River system (ZIN 14457, Tables 1 and 3), that we identify as *Lethenteron reissneri* as did Berg (1931), indicate as in nonparasitic species that ammocoete total lengths exceed those of adults, even allowing for shrinkage in spawning individuals (Vladykov and Kott 1978: fig. 12). The total lengths reported by Dybowski (1869) for the spawning adults (120–140 mm) from the upper Amur are similar to that of the spawning female from the Shangshi River (146 mm) and indicate narrow variation across a wide distributional range (Fig. 1). The absolute fecundity in 15 females collected from the Ingoda River in April–May 1969 and 1970 was estimated to be 1,720–3,360 eggs/female and the egg diameter varied between 0.68 and 0.84 mm (Karasev 1987); a low fecundity expected in a nonparasitic species (Potter et al. 2014).

The number of trunk myomeres reported for 18 adults of *Lethenteron reissneri* from Japan and Sakhalin Island by Vladykov and Kott (1978) was 57–63. This is clearly different from the counts of 70–72 based on adult specimens from the upper Amur River system (syntype) and Songhua River system (Shangshi River, Table 2) and may refer to another species. Therefore, we restrict the distribution of *Lethenteron reissneri* to the Shilka and Songhua river systems, within the Amur River basin (Fig. 1) until a more geographically comprehensive study is undertaken. Yamazaki et al. (2006) gave counts of 65–73 trunk myomeres for 55 topotypic ammocoetes from the Onon and Ingoda rivers. This is similar to the counts of 66–70 reported here (Table 3) based on two syntypes and eight topotypes.

The presence of a dark midline streak on the posterior fleshy part of the tail in ammocoetes of *Lethenteron reissneri* was distinct from the pigmentation patterns reported by Richards et al. (1982) for ammocoetes of *Entosphenus macrostomus* (Beamish, 1982), *Entosphenus tridentatus* (Gairdner in Richardson, 1836), *Lampetra ayresii* (Günther, 1870), and *Lampetra richardsoni* Vladykov & Follett, 1965. The same pattern of dark midline streak appears in Fig. 7b in Vladykov (1950) depicting the tail region of *Lethenteron appendix* (reported as *Entosphenus lamottenii*) and in Fig. 2 in Naseka et al. (2009) depicting the tail region of *Lethenteron ninae*. Additionally, Renaud et al. (in press) found the same pigmentation pattern in ammocoetes of *Lethenteron alaskense* and *Lethenteron camtschaticum*. This dark midline streak appears therefore to be a diagnostic characteristic of *Lethenteron*, but its presence still requires confirmation in *Lethenteron kessleri*.

## Diagnosis

*Lethenteron reissneri* is distinguished from parasitic *Lethenteron camtschaticum* by not feeding as an adult, reaching 182 mm compared to 625 mm TL, 5 versus 9–18 teeth on the transverse lingual lamina. It possesses 70–72 trunk myomeres in adults compared to 54–60 in *Lethenteron zanandreai* (this species has been re-assigned to the genus *Lampetra*; see Potter et al. (2015) for justification) and 58–62 in *Lethenteron ninae*; absence of pigmentation in the gular region in adults compared to presence of pigmentation in *Lethenteron appendix*; 6–7 infraoral teeth compared to 6–11 (mean 8.6) in *Lethenteron alaskense*.

*Lethenteron kessleri* (type locality: Tom’ River and tributary, Kirgizka River, Ob’ River basin, near Tomsk, Russia) is poorly defined and may be a junior synonym of *Lethenteron reissneri* as suggested by Yamazaki et al. (2006) on the basis of similarities in their number of trunk myomeres, allozyme alleles, and mtDNA genes CO I and cyt *b* sequences in larval material from the type localities (although only in a very general sense in the case of *Lethenteron kessleri*, as the material was from the upper Ob’ River basin, in the Irtysh and Uba rivers, Kazakhstan). This synonymy will require corroboration using adults and a re-examination of the former’s type material, which these authors did not conduct. It is the present authors’ intention to do so in the near future. The two undescribed *Lethenteron* spp. from Japan are distinguished from *Lethenteron reissneri* on the basis of trunk myomeres (Yamazaki et al. 2006): 49–62 (*Lethenteron* sp. S) and 51–66 (*Lethenteron* sp. N).

### Synonymy of *Lethenteron reissneri* (Dybowski, 1869) Far Eastern brook lamprey

*Petromyzon Reissneri* Dybowski, 1869: 948, 958 [original description, Onon and Ingoda rivers]

*Lampetra planeri* (non Bloch, 1784) – Berg 1906: 180 [Onon and Ingoda rivers]; Regan 1911: 203–204 [Onon River]

*Lampetra planeri reissneri* – Berg 1911: 36, 42–44 [in part, common name: Siberian brook lamprey; Siberia, Amur River basin including Sungari (Songhua) River system, Japan and eastern U. S. A.]; Berg 1916: 7 [in part, Asia and Atlantic coast of North America]

Lampetra (Lampetra) reissneri – Berg 1931: 103–105 [in part, common name: Pacific brook lamprey; Amur River basin, Pacific Ocean basin from Vladivostok to Anadyr, on Hondo (Honshu) and Hokkaido islands, Japan, possibly Pacific coast of North America]; Berg 1948: 41–43 [in part, common name: Far Eastern brook lamprey; distribution as in Berg (1931), plus Sakhalin and Iturup islands]

*Lampetra reissneri* – Nikol’sky 1956: 18–20 [in part, tributaries to upper and lower Amur River]; Abakumov 1960: 43 [in part, Amur River basin]; Hensel 1963: 85–86 [Mudan River, not Mutantiang River, tributary to Sungari (Songhua) River, People’s Republic of China]

Lampetra (Lethenteron) reissneri – Hubbs and Potter 1971: 52 [in part, distribution restricted to Asian coasts of Pacific Ocean; question raised about inconsistency between Berg’s (1948) report of usually no lower labial teeth and their observations]

*Lethenteron reissneri* – Vladykov and Kott 1978: 63 [in part, Amur River basin, Sakhalin Island and Japan, excluding Ryukyu Archipelago]; Vladykov and Kott 1979: 10, 19, 28 [in part, distribution as in Vladykov and Kott (1978)]; Kottelat 2006: 13–14 [Mongolia: Onon River system]; Yamazaki et al. 2006: 254 [Russia: Onon and Ingoda rivers, trunk myomeres in ammocoetes and adult syntype]; Bogutskaya et al. 2008: 313–314, Fig. 7 [in part, in smaller tributaries throughout Amur River basin; photograph of metamorphosing ammocoete]; Shishkin and Pavlov 2012: 429 [common name: Asiatic brook lamprey]

*Lampetra japonica* (non von Martens, 1868) – Karasev 1987: 16–18 [common name: Far Eastern brook lamprey; Ingoda, Onon, Shilka, and Argun rivers in upper Amur River basin]

### Etymology

Dybowski (1869) did not specify, but this species was possibly named after Baltic German anatomist Ernst Reissner (1824–1878), as suggested by Scharpf and Lazara (2013).

### Distribution

Shilka (Russia and Mongolia) and Songhua (People’s Republic of China) river systems within the Amur River basin (Fig. 1).

## Conclusion

This study establishes in *Lethenteron reissneri* the usual presence of a row of posterial teeth based on the examination of topotypic metamorphosing ammocoetes from the upper Amur River basin. The long and widely held perception that these teeth were either sometimes or usually absent in the species was based on the observations of Berg (1931, 1948). However, re-examination of material Berg (1931) reported to be without posterial teeth showed that these were in fact present as a complete row in the species.

*Lethenteron reissneri* possesses in adults an oral disc 3.9–5.1% of TL, a broad supraoral lamina with one cusp at either end, an endolateral tooth formula 2–2–2, an infraoral lamina with 6–7 teeth (one or both lateralmost teeth bicuspid and the rest unicuspid), 3 rows of anterials, no exolateral rows (but up to two exolateral teeth may be found on one side), 1–2 rows of posterials, the first has 24 unicuspid teeth, a transverse lingual lamina with an enlarged median cusp and two cusps on either side, a spade-like and strongly pigmented (i.e., +++) caudal fin, unpigmented (i.e., -) lateral line neuromasts and gular region, 70–72 trunk myomeres and reaches total lengths of 120–182 mm. The ammocoetes have a spade-like caudal fin, dark midline streak on the posterior fleshy part of the tail, unpigmented (i.e., -) lateral line neuromasts and lower and ventral branchial regions, weakly pigmented (i.e., +) upper lip, moderately pigmented (i.e., ++) upper branchial region, lower lip, and caudal fin, strongly pigmented (i.e., +++) cheek and area between the upper lip and cheek, a moderately pigmented (i.e., ++) bulbous middle prong of the tongue precursor and unpigmented (i.e., -) areas lateral to the elastic ridge, 65–73 trunk myomeres and reach 221.5 mm TL. It undergoes metamorphosis in August at 139.5–164 mm TL and spawns between April and June at 120–146 mm TL.
